# Breastfeeding challenges and support in a high initiation population

**DOI:** 10.1186/s13584-022-00538-5

**Published:** 2022-09-07

**Authors:** Deena R. Zimmerman, Michael Kaplan, Hanna Shoob, Marlaina Freisthler, Monique Toledano, Chen Stein-Zamir

**Affiliations:** 1 Department of Maternal and Child Health, Public Health Services, Ministry of Health, 39 Yirmyahu St, P.O.B. 1176, 9446724 Jerusalem, Israel; 2 Jerusalem District Health Office, Ministry of Health, 86 Jaffa Road, Jerusalem, Israel; 3 Faculty of Medicine, The Hebrew University and Hadassah Braun School of Public and Community Medicine, The Hebrew University of Jerusalem, Jerusalem, Israel

**Keywords:** Breastfeeding, Support, Initiation, Continuation, Associated factors

## Abstract

**Background:**

The breastfeeding initiation rate in Israel is approximately 90%, yet exclusive breastfeeding drops sharply in the early postnatal period. The study objective was to assess early postpartum professional breastfeeding support, its association with breastfeeding success and identification of risk factors for early breastfeeding discontinuation.

**Methods:**

As part of a post-discharge newborn follow-up study, a convenience sample of 868 mothers attending Maternal and Child Health Centers (MCHCs) up to three months post-partum were interviewed using a 26-item questionnaire. Breastfeeding-related questions covered demographic variables, pregnancy and birth details; breastfeeding duration, lactation support in hospital and post-discharge; and problems experienced.

**Results:**

Most mothers, 797 (91.8%), initiated breastfeeding in hospital. All women who initiated breastfeeding in the hospital reported exclusive breastfeeding; by two weeks postpartum, 70 women (13.2%) were supplementing with formula (partial breastfeeding). Kaplan–Meier Survival Analysis revealed an estimated mean duration of exclusive breastfeeding in the sample population of 66.8 ± 1.5 days. This duration was shorter for women with preterm births, low birthweight infants (LBW), cesarean births, and hospitalizations in neonatal intensive care units (NICU). A total of 472 (59.3%) breastfeeding mothers reported receiving in-hospital guidance. Of these, 290 (61.3%) were observed breastfeeding. Of all women who initiated breastfeeding, 280 (35.1%) attended MCHC follow-up within 72 h of hospital discharge. A higher proportion of women experiencing breastfeeding difficulties attended an MCHC within 72 h (131/297, 44.1%) compared to women not experiencing difficulties (148/499, 29.7%). The most frequently reported problems were mechanical (55.2%) or milk supply concerns (18.5%). First-time mothers were more likely to report problems, as were Jewish (vs Arab) mothers.

**Conclusions:**

Even in a population with high initiation rates of breastfeeding, breastfeeding duration, both exclusive and partial, is less than recommended. As much of this drop-off occurs during maternity leave, it is likely related to breastfeeding challenges other than employment. Breastfeeding support needs of women are currently not adequately met; staffing and time for both in-hospital and community-based counseling needs to be funded as well as mandated. Counseling hours should be tailored to assure adequate coverage of high-risk groups such as women after cesarean delivery and newborns requiring intensive care.

## Background

Breastfeeding has been shown to provide health benefits for both mother and child [1–4]. Israel's Ministry of Health (MOH) has adopted the World Health Organization’s recommendation for exclusive breastfeeding for the first six months of life [5]. Following the approach of the American Academy of Pediatrics [6], the MOH recommends continued breastfeeding for a minimum of one year and for as long as mutually desired by mother and child [7].

In practice, rates of breastfeeding initiation and continuation of exclusive or partial breastfeeding vary wordwide [8]. Many factors are thought to influence breastfeeding rates and can be grouped into pre-conception, antenatal, perinatal and postpartum factors [9]. Often, the perinatal factors cited by women as reasons for early cessation of breastfeeding are amenable to interventions. These factors include, for example, mechanical breastfeeding problems, such as trouble with latching-on or painful nipples, as well as concerns over milk supply [10]. Effectively addressing these issues in the perinatal period has the potential to help prolong the breastfeeding relationship.

Israel has a breastfeeding initiation rate of approximately 90% [11]. Currently, comprehensive comparative initiation statistics for high-income countries are not available. Data from three OECD (Organisation for Economic Co-operation and Development) countries show that Israel’s initiation rate is lower than Norway, but higher than the United States [12]. The rate of exclusive breastfeeding in Israel drops sharply within the first months after birth. By two months after birth, only about 58% of women are exclusively breastfeeding and by six months, only 20% [11]. Several other high-income countries with high initiation rates, such as Australia, Canada, Germany, Norway, and New Zealand, have reported similar declines in breastfeeding rates at three and four months after birth. Other countries (e.g., the United Kingdom, the Republic of Ireland, the United States, and France) report even lower rates of breastfeeding at the 3–4 month time period, but this is usually coupled with lower initiation rates [13]. This common trend is concerning, given the growing information regarding the importance of breastfeeding to maternal and child health.

The Israeli health system has a number of strengths that support breastfeeding. All citizens of Israel are covered by health insurance, which is provided by one of four health funds. MOH directives mandate that all mothers should be offered a breastfeeding assessment by a breastfeeding counselor within the first 24 h after birth and at least once daily for the duration of their hospital stay. Breastfeeding counselors, who are nurses who have received training in accordance with MOH requirements, are able to assess complications that arise and provide assistance to mothers as needed [14]. Because more than 99% of births in Israel occur in a hospital, universal support should thus be available [15]. Well-baby care in Israel is provided by MCHCs [16]. When mothers leave the hospital, they are instructed to bring their newborns to their neighborhood MCHC for a follow-up evaluation by a public health nurse or their health fund physician, within 72 h of discharge [17]. MOH directives indicate that all public health nurses should have lactation training, providing additional timely support [14].

The data reported in this analysis are based on a subset of questions from a larger study whose purpose was to evaluate adherence to newborn discharge instructions [17]. The goal of this subset of questions was to assess whether women received professional breastfeeding support, and to measure their level of interaction with the health care system. Additional aims were to analyze associations between the support received by women and their breastfeeding success in the early post-natal period, and to identify risk factors of early discontinuation of breastfeeding in our study sample. This community-based study aims to provide information for planning effective interventions to improve the sustainability of breastfeeding during the postpartum period.

## Methods

### Setting

At the time of this study, the Jerusalem District’s population was approximately 1.2 million, representing around 14% of Israel’s total population. About 72.3% of the population was Jewish (secular/traditional 49%, and ultra-Orthodox 12%), while 27.7% of the population was Arab (primarily Muslim). There were, on average 32,600 births per year in this district, representing 18.5% of the total births in Israel [18].

### Population

A convenience sample included mothers of infants < 3 months of age attending one of 14 MCHC facilities in the Jerusalem District between January 2017 and February 2018. To avoid overrepresentation of any specific subgroup, the number of study participants included in each subgroup was modified to keep the proportion similar to the district’s population distribution.

### Design

The study involved face-to-face interviews of mothers conducted by a single research assistant trained in performing interviews of this nature.

### Instrument

A structured 26-item questionnaire, designed for a study of newborn discharge follow-up, was administered [17]. The subset reported in this study include the items (1) demographic variables of mother and child; (2) information regarding the pregnancy and birth; (3) details about lactation support received in the hospital and following discharge; (4) timing and place of first evaluation; (5) reasons for non-follow up at a MCHC within 72 h of discharge; (6) problems experienced during breastfeeding; (7) infant’s weight changes during the post-natal time period.

### Sample-size calculation

Distribution of responses were designated into two main groups by two degrees of “agree” versus “disagree” leading to a 50/50 probability. Assuming a margin of error of 5% and a confidence interval of 95%, the sample size was calculated. The sample size needed to represent 32,000 deliveries annually with a 95% confidence interval and a 3.5–4.0% margin of error was calculated to be between 589 and 765 newborns. We increased the desired number to 800 to ensure adequate representation of population subgroups.

### Main outcomes measured and definitions

The main outcome measures were length of exclusive breastfeeding and length of partial breastfeeding.

### Statistical Analysis

Descriptive statistics were evaluated with respect to demographic variables and questionnaire responses. Differences in continuous variables were analyzed using Student’s T-Test and Wilcoxon Rank Sum Test, while differences in proportions for categorical variables were analyzed using Pearson’s Chi-squared Test. Kaplan Meier (KM) curves with Log Rank Tests to discern differences between groups were used to describe the "survival time" of breastfeeding (exclusive and partial) and probability of breastfeeding cessation, and to compare stratified "survival curves" based on relevant variables. Univariate and multivariable Cox-Proportional Hazards Regression were used to model associations, using backward elimination. Both crude and adjusted hazard ratios (HR) with calculated 95% confidence intervals were reported. Two-sided p-values of less than 0.05 were considered to be statistically significant. Analyses were performed in SAS (Statistical Analysis System) 9.4 (SAS Institute, Cary, NC).

## Results

There were 873 mothers approached during the study period. Of those, 868 (99.4%) agreed to be interviewed. Of the women who were interviewed, 797 (91.8%) reported initiating breastfeeding in the hospital after birth. Three additional women reported initiation of breastfeeding sometime after discharge from the hospital for a total of 800 (92.2%) women who initiated breastfeeding. Complete data was available for 796 of the 797 women who reported initiating breastfeeding in the hospital after birth. Demographic variables for the study population and factors relating to the pregnancy, birth and hospital stay by groupings of women who reported initiating breastfeeding compared to those who reported not initiating breastfeeding are presented in Table 1.

**Table 1:** Study Population by Grouping of Breastfeeding Initiation or Not

Variable	Initiated	Did Not Initiate	p-value*
*Mother*			
Overall (n = 796)			
Age	29.5 (± 5.5)	31.0 (± 5.5)	0.023
Number of Children	2.4 (± 1.6)	2.6 (± 1.4)	0.127
Maternal Education			< .0001
High School	292 (87.4%)	42 (12.6%)	
College	486 (94.9%)	26 (5.1%)	
Nationality			0.6401
Israeli-Born	666 (91.7%)	60 (8.3%)	
Not Israeli-Born	131 (92.9%)	10 (7.1%)	
Ethnicity/Religiosity			0.0004
Arab	155 (97.5%)	4 (2.5%)	
Jewish—ultra-Orthodox	135 (96.4%)	5 (3.6%)	
Jewish—Trad./Sec	505 (89.4%)	60 (10.6%)	
*Birth and Baby*			
Average Gestation	39.1 (± 1.72)	38.5 (± 1.92)	0.0073
Hospital Length of Stay	3.6 (± 4.6)	3.8 (± 3.0)	0.1616
Pregnancy			0.029
Complicated	41 (83.7%)	8 (16.3%)	
Uncomplicated	756 (92.4%)	62 (7.6%)	
Birth			0.0002
Vaginal	666 (93.5%)	46 (6.5%)	
Cesarean	131 (84.5%)	24 (15.5%)	
Preterm			0.0116
Yes	44 (83.0%)	9 (17.0%)	
No	747 (92.7%)	59 (7.3%)	
Sex			0.7024
Male	383 (92.5%)	31 (7.5%)	
Female	392 (91.8%)	35 (8.2%)	
Hospitalization			< .0001
Nursery	651 (92.7%)	51 (7.3%)	
Partial Rooming In	13 (92.9%)	1 (7.1%)	
Full Rooming In	85 (96.5%)	3 (3.4%)	
NICU	26 (70.3%)	11 (29.7%)	

*Student’s T-Test and Wilcoxon Rank Sum Test used to measure differences in continuous variables. Pearson’s Chi-squared Test used to measure differences in categorical variables

Women who reported initiating breastfeeding were on average younger than those who reported not initiating. Women who had completed at least a bachelor’s degree were more likely to report initiating breastfeeding than those whose education ended after high school. Arab women were more likely to report initiation of breastfeeding compared to Jewish women. Among Jewish women, ultra-Orthodox women were more likely to initiate breastfeeding than traditional/secular Jewish women.

Lack of breastfeeding initiation was significantly associated with cesarean births vs. vaginal deliveries, complicated pregnancies, preterm births, and NICU admission. There was no association with initiation of breastfeeding for gender of baby, number of children (categorical), first child, birthplace of mother, and birthweight.

### Breastfeeding Support

Table 2 presents descriptive data for the study participants by whether or not they experienced problems with breastfeeding. Of the mothers who reported initiating breastfeeding in the hospital, 472 (59.3%) reported receiving breastfeeding guidance during their hospital stay. Of these mothers, 290 women (61.3%) reported that a health care professional had directly observed a breastfeeding session during their hospital stay. Higher rates of both breastfeeding consultation (87.9% vs. 42.3%, p < 0.0001) and observation (63.6% vs. 20.3%, p < 0.0001) were reported among women who described problems with breastfeeding versus those who did not.

**Table 2:** Descriptive Variables for Groups by Experiencing Problems or Not

Variable	Experienced Problems Breastfeeding		p-value*
	No	Yes	
Overall (n = 796)	499 (62.6%)	297 (37.3%)	
Parity			< 0.0001
First-Time Mother	103 (37.3%)	173 (62.7%)	
Veteran Mother	396 (76.1%)	124 (23.8%)	
Ethnicity/Religiosity			0.0012
Arab	113 (72.9%)	42 (27.1%)	
Jewish—ultra-Orthodox	92 (68.1%)	43 (31.9%)	
Jewish—Trad/Sec	292 (57.9%)	212 (42.1%)	
Preterm			0.0021
No	477 (63.9%)	269 (36.1%)	
Yes	18 (40.9%)	26 (59.1%)	
Birthweight			< 0.0001
LBW	19 (35.8%)	34 (64.2%)	
Normal	480 (64.6%)	263 (35.4%)	
Hospitalization			0.0108
Nursery	414 (63.6%)	237 (36.4%)	
Rooming In	62 (63.9%)	35 (36.1%)	
NICU	9 (34.6%)	17 (65.4%)	
Lactation Counseling (Hospital)			< 0.0001
Breastfeeding consultant	156 (40.3%)	231 (59.7%)	
Nurse	55 (64.7%)	30 (35.3%)	
None	288 (88.9%)	36 (11.1%)	
Observed Breastfeeding			< 0.0001
Yes	101 (34.8%)	189 (65.2%)	
No	396 (78.6%)	108 (21.4%)	
MCHC within 72 h			< 0.0001
Yes	148 (53.0%)	131 (47.0%)	
No	351 (67.9%)	166 (32.1%)	
Lactation Counseling at MCHC			< 0.0001
Yes	15 (23.4%)	49 (76.6%)	
No	484 (66.1%)	248 (33.9%)	

*Student’s T-Test and Wilcoxon Rank Sum Test used to measure differences in continuous variables. Pearson’s Chi-squared Test used to measure differences in categorical variables

Of the women who received an inpatient breastfeeding consultation (n = 472), breastfeeding support was provided by a breastfeeding consultant for most (82%). For the others, counseling was provided by a departmental nurse (18%). Women who were experiencing problems (n = 297) were more likely to receive a consultation with a breastfeeding consultant (59.7%) rather than a consultation with a departmental nurse (35.3%) or no consultation (11.1%) (p < 0.0001).

Among all the women who initiated breastfeeding, 64 (8.0%) received a breastfeeding consultation from a public health nurse with lactation training at the first MCHC visit. Women who were experiencing breastfeeding difficulties were more likely to receive these consultations (49/297, 16.5%, p < 0.0001). There were no significant associations between women who received the first visit consultation with respect to age, number of children, parity, ethnicity, or religiosity of the mother.

Of the women who initiated breastfeeding in the hospital, 279 (35.1%) attended an MCHC visit within 72 h of hospital discharge. Out of 297 women experiencing breastfeeding problems, 131 (44.1%) attended MCHC within 72 h, whereas out of 499 not experiencing problems, 148 attended within 72 h (29.7%) (p < 0.0001). Of the 131 women who were experiencing problems who went to MCHC within 72 h, 23 (17.6%) women had a lactation consultation at their visit.

### Breastfeeding Difficulties

Breastfeeding difficulties were reported by 297 (37.3%) of the women who initiated breastfeeding. The most frequent types of problems reported were mechanical problems with breastfeeding (55.2%) and problems with milk supply (18.5%). Other problems reported included difficulty for mother (9.8%), mother not interested in breastfeeding (8.4%), and illness of the infant (3.4%). Problems were significantly more common among first-time mothers. Jewish women were more likely to report problems compared to Arab women (39.9% vs. 27.1%, p = 0.0031). Traditional/secular Jewish women were more likely to report problems compared to ultra-Orthodox Jewish women (42.1% vs. 31.9%, p = 0.0314). Mothers of preterm babies were more likely to report problems compared to mothers of term babies. Mothers of low birthweight babies were more likely to report problems with breastfeeding than mothers of babies with normal birthweight. Similarly, there was an association between NICU admission and problems breastfeeding. There were no significant associations between problems with breastfeeding and mother’s country of birth, education level, pregnancy complications, and type of delivery.

Information regarding weight loss and supplementation, based on the mothers' reports from their hospital stay, was analyzed. A total of 76 newborns (9.5%) of mothers who wished to exclusively breastfeed received supplementation. Of these, 39 mothers reported excessive weight loss in their infants. The percent change in weight of the infants who received supplementation was not statistically significantly different from the percent change in weight of those infants who did not receive supplementation. For many of these infants, the degree of weight loss was less than that which would be considered a medical indication for supplementation [19].

### Breastfeeding Duration

**Fig. 1:**
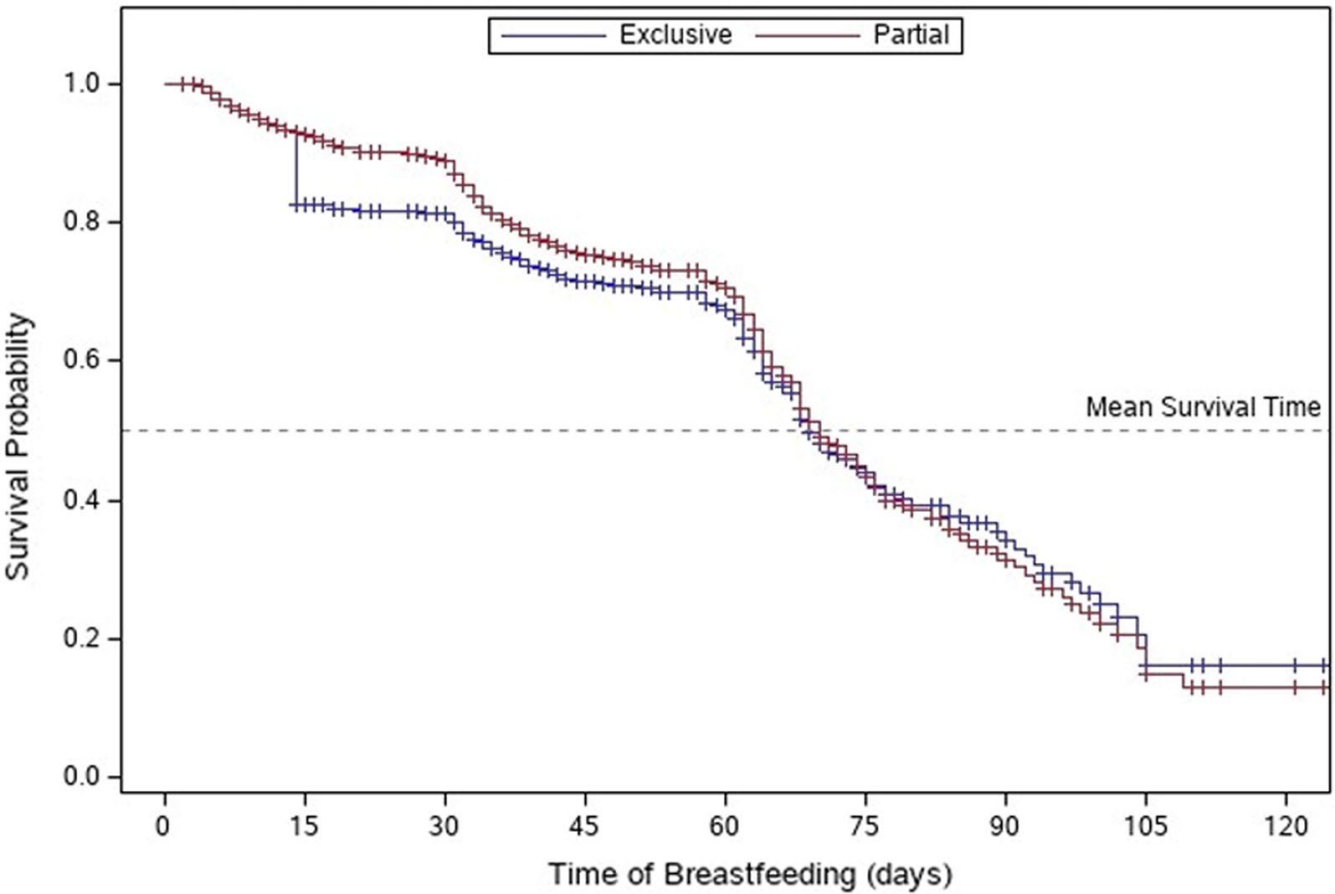
Survival Analysis ofBreastfeeding Among Initiators. Kaplan Meier (KM) curves with Log Rank Tests were used to discern differences between groups described as "survival time" of breastfeeding (exclusive and partial)

At the time of the survey, infants were between a few days old up to three months of age. For mothers whose infants were at least two weeks old at the time of the survey (n = 629), all who initiated breastfeeding in the hospital reported continued breastfeeding, although some were using supplements. Because of the varying age of infants at the time of the survey, KM Method was used to estimate mean survival (i.e., duration) of both exclusive breastfeeding, and any level of breastfeeding (Fig. 1). Women who initiated breastfeeding after discharge from the hospital were not included in this analysis. The estimated mean duration of exclusive breastfeeding in the sample population was 66.8 ± 1.5 days, and the estimated mean duration of partial breastfeeding was 69.7 ± 1.5 days.

**Fig. 2:**
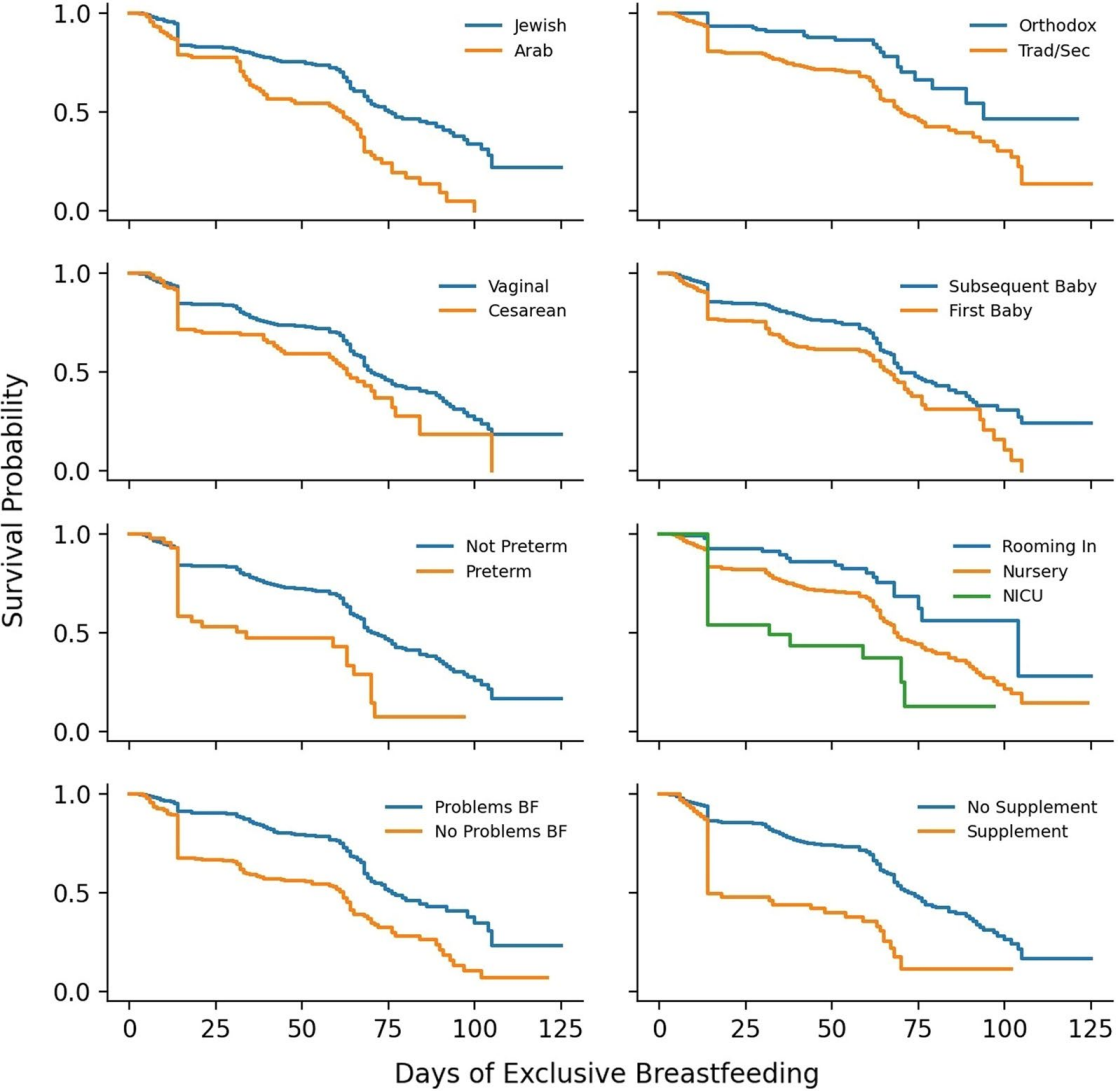
Breastfeeding Survival Estimates. Univariate survival analysis using Cox-Proportional Hazards Regression was performed to analyze differences in survival probability of exclusive breastfeeding in the first few months after birth

Univariate survival analysis was performed to analyze differences in survival probability of exclusive breastfeeding in the first few months after birth (Fig. 2). The difference was statistically significant for breastfeeding survival (i.e., duration) between women who were first time mothers and veteran mothers, women who were not college-educated and those who were, Arab women compared to Jewish women, and traditional/secular Jewish women compared to ultra-Orthodox Jewish women.

Problems during pregnancy and childbirth appeared to influence the duration of breastfeeding as well. The estimated mean duration of breastfeeding was shorter and found statistically significant for women having a preterm birth, having an infant birth weight of less than 2500 g, having a cesarean birth, and having a baby hospitalized in NICU. Mothers who were able to room-in during hospitalization had a longer estimated mean duration of breastfeeding compared to mothers whose babies were admitted to the infant ward or NICU.

Experiencing problems with breastfeeding was also an important factor influencing breastfeeding duration. Women who experienced problems with breastfeeding had a shorter duration of breastfeeding compared to women who did not experience problems. Women who reported that their infants experienced excessive weight loss also had a shorter mean duration of breastfeeding, as did women who reported that their newborn required supplementation in hospital.

Although women who received a breastfeeding consultation had a significantly shorter mean duration of breastfeeding (63.14 ± 2.01 vs. 72.07 ± 2.34), as did women who were clinically observed breastfeeding during their breastfeeding consultation (63.06 ± 2.65 vs. 68.82 ± 1.89), this appears to be a proxy for women with breastfeeding difficulties. Differences in duration of breastfeeding with respect to mother’s country of birth, concerns for jaundice in the hospital, requiring bilirubin testing or phototherapy, experiencing pregnancy complications, change in weight from birth to discharge, percent change in weight from birth to discharge, receiving a breastfeeding consultation at MCHC, or coming to MCHC within 72 h after discharge were not statistically significant.

An Extended Cox Regression Model was used to evaluate the effects of selected variables on duration of breastfeeding. The following variables identified as predictors: problems with breastfeeding (p < 0.0001), stratified ethnicity and religiosity (traditional/secular Jewish vs. Arab (p < 0.0001); vs. ultra-Orthodox Jewish (p < 0.0001)), gestational age (p = 0.0214), type of hospitalization (rooming-In vs. not, p = 0.0015), and supplementation in-hospital (p = 0.0016) and were found to have a statistically significant effect on duration of breastfeeding. Experiencing problems breastfeeding was determined to be a time-dependent variable requiring the addition of an interaction term (p = 0.0120).

Compared to identifying as traditional/secular Jewish, identifying as Arab was a positive predictor of hazard of discontinuing breastfeeding, whereas identifying as ultra-Orthodox was a negative predictor of hazard. Increased length of gestation was a negative predictor of hazard of discontinuing breastfeeding. Not rooming in, receiving supplementation, and experiencing breastfeeding problems were all positive predictors of hazard. The interaction effect of breastfeeding and time indicated that, although breastfeeding problems were a positive predictor of hazard of discontinuation of breastfeeding, the hazard of discontinuation decreased with time. That is, experiencing breastfeeding problems (vs. not experiencing problems) was a time dependent positive predictor of hazard, with hazard decreasing as time passed since birth.

The parameter estimates of the fitted model can be interpreted as follows: A one-day increase in length of gestation resulted in an estimated 7.2% (CI 1.1–12.8) decrease in hazard for discontinuation of breastfeeding. Arab women had an estimated 1.85 (1.41–2.42) times the risk of discontinuation of breastfeeding compared to traditional/secular Jewish women, whereas the risk for ultra-Orthodox Jewish women was 0.42 (0.28–0.65) times as much as that for traditional/secular Jewish women. Women who did not room-in with their babies had 2.17 (1.36–3.48) times the risk of discontinuation of breastfeeding compared to women whose babies did room-in. Women whose babies received supplementation had an estimated 1.82 (1.25–2.63) times the risk of discontinuation of breastfeeding compared to women whose babies did not receive supplementation. The hazard ratio for discontinuation of breastfeeding due to problems breastfeeding decreases over time. The effect of the interaction term is summarized in Table 3.

**Table 3:** Effect of Measured Variables on Duration of Breastfeeding

Variable	Parameter Estimate*	SE	χ^2^	DF	p-value	Hazard Ratio*	95% CI
Gestational Age	− 0.07422	0.0322	5.2914	1	0.0214	0.93	0.87–0.99
Ethnicity/Religiosity (“Arab”)	0.61371	0.13756	19.9054	1	< 0.0001	1.85	1.41–2.42
Ethnicity/Religiosity (“Orthodox Jewish”)	− 0.85811	0.21665	15.6883	1	0.0001	0.42	0.28–0.65
Hospitalization (“Other”)	0.77583	0.23972	10.4746	1	0.0015	2.17	1.36–3.48
Supplement	0.59710	0.18937	9.9425	1	0.0016	1.82	1.25–2.63
Breastfeeding Problems	1.22357	0.22271	30.1829	1	< 0.0001	3.40	2.20–5.26
Breastfeeding Problems * Time	− 0.01196	0.00476	6.3040	1	0.0120	0.99	0.98–1.00

Gestational age = gestational age at time of birth in days; Ethnicity/Religiosity = effect of identifying as Arab (compared to Traditional/Secular Jewish) or Orthodox Jewish (compared to Traditional/Secular Jewish); Hospitalization = effect of not rooming in (compared to rooming in); Supplement = effect of receiving supplementation (compared to not receiving supplementation); Breastfeeding Problems = effect of reporting experiencing problems with breastfeeding (compared to not reporting problems with breastfeeding); and Breastfeeding Problems * Time = interaction effect of time and breastfeeding problems

*Extended Cox Regression was used to determine parameter estimates and hazard ratios

## Discussion

Even in a population with high initiation rates of breastfeeding, breastfeeding duration, both exclusive and partial, is less than the MOH recommendations. As much of this drop-off occurs during maternity leave, it is likely to be related to breastfeeding challenges other than employment. Early breastfeeding challenges such as pain during breastfeeding and perceived inadequate intake are amenable to professional support. Health policies are an important step to encourage such support, but it is important to ensure that polices are being successfully implemented. The study findings indicate that the goals of the MOH directives are currently not being met. Approximately half of women studied reported not receiving breastfeeding support during their hospitalization. Women in our survey were not asked why they did not receive this support, but a lack of adequate staffing to fulfill the directives may be the cause. This lack of support is of clinical importance as proper breastfeeding initiation practices and access to post-discharge professional breastfeeding support is recognized as crucial to breastfeeding success [10, 20–23].

Within the limited service provided at present, however, it appears that care is being effectively triaged. The vast majority of women who were experiencing problems with breastfeeding were seen by a breastfeeding counselor in the hospital, both in the intensive care unit and the general ward. Women experiencing difficulties were also significantly more likely to receive outpatient help in an MCHC. The breastfeeding difficulties experienced by women in this study were similar to those reported in a previous study on breastfeeding in Israel [11]. The majority of problems experienced by these women are amenable to intervention, which stresses the need for providing early support. It is also important to provide universal support to identify situations where the mother does not perceive a problem until a point where harder to remedy.

Of the women reporting on problems experienced with breastfeeding, nearly one in five (18.5%) reported concerns about milk production, a concern commonly cited in the literature [24–26]. Given that the volume requirement of newborn infants is minuscule, it is likely that most of these mothers produced the required amount during the 36–48 h of hospitalization. A need for additional education, particularly for new mothers regarding the normal and expected progression of breastmilk production is shown in accordance with the literature cited [23–25].

Nearly 10% of the “exclusively breastfeed” infants in this study received supplementation, including more than 5% of babies for whom there was no concern about weight loss. Given the known detrimental effects of unnecessary supplementation [26], as well as the observed negative effects demonstrated in an isolated survival analysis and regression modeling, instruction for hospital staff regarding the appropriate use of supplementation can be a useful intervention to extend exclusive breastfeeding among women in Israel.

One issue of particular concern is how mothers of at-risk neonates are being supported in their attempts to breastfeed. In this study sample, preterm infants, low birthweight infants, and infants admitted to the NICU all demonstrated reduced breastfeeding duration. There were 87 women in the study whose newborns were in these groups. In this high risk population, only 62/87 (71.3%) had a consultation. Maternal complaint of difficulties was more likely to lead to consultation. Within this at-risk group, 47 mothers reported having breastfeeding difficulties. Among them, 39 (83.0%) had a consultation and 8 (17.0%) did not have a consultation.

When controlled for other factors, gestational age had a significant effect on risk for discontinuation of breastfeeding. As reduced breastfeeding rates are often seen in preterm infants [27, 28], this finding is not surprising. This population would benefit from special attention such as assuring professional in hospital and post-discharge consultation for all premature. The clear benefit of exclusive breastfeeding for preterm infants warrants careful consideration to determine where the current model falls short.

Previous research has demonstrated high rates of breastfeeding initiation and extended duration among both Arab and Jewish ultra-Orthodox women in Israel [11]. While breastfeeding discontinuation was low among Jewish ultra-Orthodox women in this study compared to traditional/secular Jewish women (as expected), breastfeeding duration was lower among Arab women than expected. This research suggests that breastfeeding duration may not be as consistent among Arab women, at least in the Jerusalem District, as previously demonstrated. However, the sample size of Arab women in this study is substantially smaller than the sample size of Arab women in the previous research on breastfeeding in Israel [11] which might explain the discordance in results. Nonetheless, this study raises concerns about the effectiveness of breastfeeding support for Arab women in the Jerusalem community. Adapting guidelines for improving breastfeeding support in this population should be considered.

This study also indicates that access to breastfeeding support available for women during the postpartum period is inadequate. Attendance of women to MCHCs during the first few days after hospital discharge is not uniform. When follow-up care is received at MCHCs, only a small percentage of women are offered breastfeeding-specific counseling sessions. The need for development of an intervention to improve follow-up care rates during this vulnerable time period has been revealed and steps should be taken to augment the MCHC services to better meet the breastfeeding support needs of women with newborns. During the COVID-19 Pandemic, many MCHCs began to offer online support. Adoption of this model may be one way to increase routine services.

### Limitations

Study findings are based on patient reporting and are not the results of direct observation. The use of a convenience sample in a circumscribed area of the country limits the generalizability of the findings. The retrospective nature of the study design could lead to recall bias. Despite these limitations, this study provides important data that can be used towards the design of future studies.

## Conclusion

While breastfeeding initiation rates in Israel are exceptionally high, exclusive breastfeeding rates drop quickly over the first few weeks after birth. Theoretically, there is an extensive system of support for breastfeeding women within the Israeli healthcare system during this critical time period, however, in practice the breastfeeding support needs of many women are currently not being adequately met. Funding is needed to assure that trained personnel exists in practice and has the time, protected from other duties, to provide the needed education and guidance to new mothers. Counseling hours should be tailored to assure adequate coverage of high risk groups such as women after cesarean delivery and newborns requiring care in the neonatal intensive care unit. The majority of problems experienced by women in this study are amenable to intervention, which stresses the need for providing early and universal support. Assuring adequate education of all health care professionals working with breastfeeding dyads regarding breastfeeding physiology can reduce inappropriate supplementation. Funding for adequate staffing in MCHCs to assure timely continuity of care and expanding extended hours of post-discharge support can help build the skills and confidence of the mothers that aid successful lactation. Building such a constellation of support is what is likely needed to reach recommended duration of exclusive breastfeeding.

## Data Availability

All data supporting the results reported in the article can be provided upon request.

## Abbreviations

MCHCs: Maternal and Child Health Centers
LBW: Low birthweight infants
NICU: Neonatal intensive care units
MOH: Ministry of Health
OECD: Organisation for Economic Co-operation and Development
KM: Kaplan Meier
HR: Hazard ratios
SAS: Statistical Analysis System
